# Liesegang-Like Rings in Lactational Changes in the Breast

**DOI:** 10.1155/2012/268903

**Published:** 2012-10-10

**Authors:** Mohd T. Islam, Joyce J. Ou, Katrine Hansen, Rochelle A. Simon, M. Ruhul Quddus

**Affiliations:** Department of Pathology, The Warren Alpert Medical School of Brown University and Women & Infants Hospital, 101 Dudley Street Providence, RI 02905, USA

## Abstract

Liesagang-like rings (LR) are periodic structures with equally spaced radial striations formed by a process that involves diffusion, nucleation, flocculation or precipitation, and supersaturation. Being more common *in vitro*, on rare occasions also reported *in vivo* in association with inflammatory or cystic lesions and confused with parasites or calcification on needle aspirates. The current paper documents that LRs may be seen in noncystic and noninflammatory changes of the breast.

## 1. Introduction

Liesegang rings (LRs) are periodic precipitation zones from supersaturated solutions in colloidal systems. The LRs are possibly formed by a process that involves diffusion, nucleation, flocculation or precipitation, and supersaturation. Notable examples include LRs of calcium carbonate in oölitic limestone (in nature), silver chromate in gelatin (*in vitro*), and LRs of glycoprotein in pulmonary corpora amylacea (*in vivo*) [1]. The LRs vary greatly in shape and size, measuring 7–800 microns.

Being more commonly seen *in vitro*, LRs have also rarely been reported *in vivo*—mostly in association with lesions of the kidney and synovium, however, their presence has also been described in the conjunctiva, eyelid, paranasal sinus, peritoneum, and in association with endometriosis [1–3]. Isolated cases of LRs have been reported associated with breast cysts and mammary duct ectasia [4, 5]. 

These rings usually form in association with cystic, fibrotic, inflamed, or necrotic tissues, and may be mistaken for eggs, larvae, or parasites.

We report here a case of Liesegang rings in lactational change of the breast, noted incidentally in a mastectomy specimen for breast carcinoma.

## 2. Case Report

A 39-year-old, G0, Hispanic woman presented with a mass in her right breast. The patient denied the use of hormone replacement therapy, birth control pills, or substance abuse in the past. She had never conceived naturally, had failed *in vitro* fertilization, and had never breast-fed. No family history of breast, ovarian, uterine, or colon carcinoma was present. An axillary ultrasound revealed an enlarged abnormal axillary lymph node. An ultrasound-guided core needle biopsy of the breast mass revealed a moderately differentiated infiltrating duct carcinoma. 

**Figure 1:**
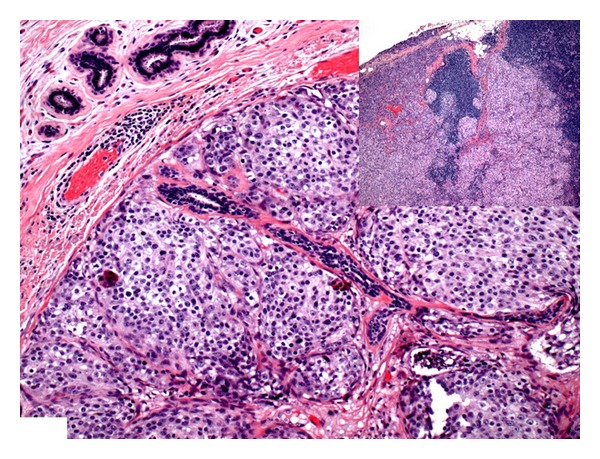
Infiltrating duct carcinoma, Nottingham grade 2 of 3. Inset: Metastatic carcinoma in the axillary lymph node (H&E; original ×20).

The patient opted for a simple mastectomy of the right breast, and right axillary sentinel lymph node sampling was performed. The final pathology revealed a 2.2 cm, grade 2, hormone receptor positive, Her2/neu negative, infiltrating duct carcinoma (Figure 1), with associated grade 2 *in situ* carcinoma and negative margins. One of 5 sentinel lymph nodes showed a 0.5 cm metastatic tumor deposit with extracapsular extension (Figure 1). She received systemic chemotherapy after surgery.

**Figure 2:**
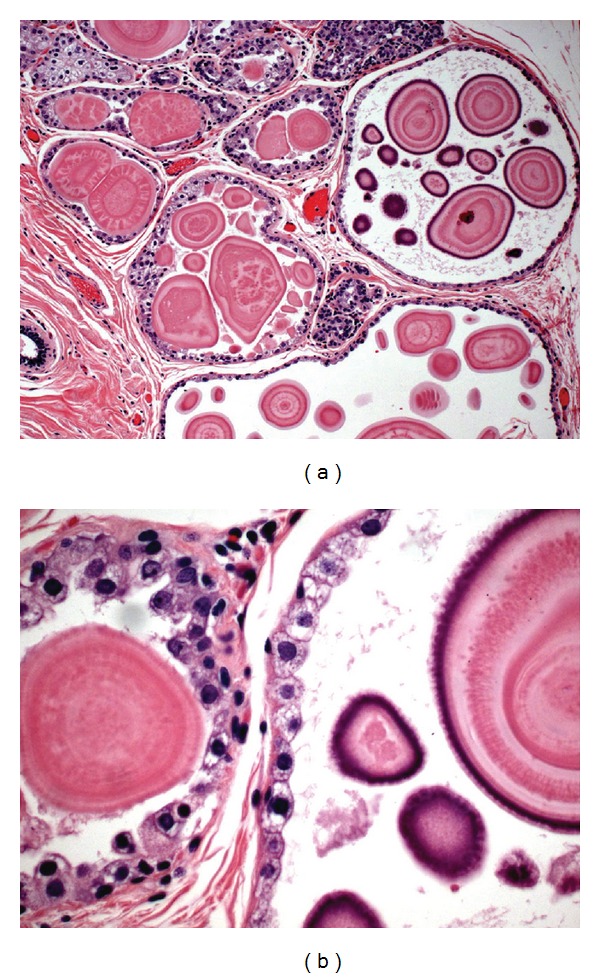
(a) Liesegang-like rings (LRs) in slightly dilated breast ducts associated with lactational change (H&E; original ×10). (b) Higher magnification of Liesegang-like rings and lactational change (H&E; original ×40).

In addition to invasive and *in situ* breast carcinoma, the breast tissue showed proliferative fibrocystic changes as well as lactational changes. In areas of lactational change, focal dilated glands contained intraluminal, acellular structures with equally spaced radial striations; these were identified as Liesegang rings (Figure 2).

## 3. Discussion and Conclusions

The formation of Liesegang rings is a well-known phenomenon in the field of chemistry, occurring most commonly *in vitro*. It was first described by German biochemist Ralph *E.* Liesegang as a process involving the formation of periodic precipitation zones around a central nidus. These precipitation zones are seen microscopically as concentric laminations and are formed via alternating cycles of subsaturation and supersaturation of an insoluble product produced by diffusing reactants within a colloidal matrix or gel [6]. These characteristic Liesegang rings are rarely seen *in vivo*, but when identified are usually found in association with cystic or inflammatory lesions. They may be mistaken for parasites on fine-needle aspiration or surgical specimens of hemorrhagic areas.

The exact composition of Liesegang rings is not fully understood. LRs were initially believed to represent parasites, in particular adult forms of the giant kidney worm* Dioctophyma renale* (a large blood-red nematode that infects a variety of fish-eating mammals, especially mink) [1]. The rings may range from 7 to 800 microns in diameter and have uniform, pink-tan, radially striated, double walls. Multiple small rings may also be seen within a larger, dominant ring. Raso et al. [6] noted that LRs displayed some morphological differences from *D. renale* when compared with specimens from animals infected naturally or experimentally with the giant kidney worm, as they lack the internal organs of such parasites and the histopathologic changes associated with such infections. Histochemical and immunoperoxidase stains for mucopolysaccharides, amyloid, and keratin are reportedly negative [6]. However, results for calcium, iron, and hemoglobin vary in the literature [1, 6, 7], suggesting that *in vivo*, local environmental factors may create LRs of differing compositions. Special stains and energy-dispersive radiographic analysis or scanning electron microscopy have revealed that some LRs also contain silicon and sulfur [1], while ultrastructural analysis has revealed an electron dense core and fine fibrillary rings with a concentric and radial pattern [6, 8].

Liesegang rings are rarely present in biological systems; therefore their presence in cystic and inflamed tissues may be confused with various parasites, algae, calcifications, corpora amylacea, psammoma bodies, and the spheroid type of amyloid. In the current paper, LRs were found in association with slightly dilated breast ducts, with the epithelium showing changes similar to those seen in lactation; an incidental finding in a patient with AJCC stage IIB breast carcinoma. The current case documents that LRs may also be found in noncystic and noninflammatory conditions in the breast.

## References

[1] Tuur SM, Nelson AM, Gibson DW (1987). Liesegang rings in tissue: how to distinguish Liesegang rings from the giant kidney worm, Dioctophyma renale. *American Journal of Surgical Pathology*.

[2] Sneige N, Batsakis JG, Hawkins RA, Doble HP (1988). Pseudoparasitic (Liesegang) bodies in paranasal sinus. *Journal of Laryngology and Otology*.

[3] Clement PB, Young RH, Scully RE (1989). Liesegang rings in the female genital tract. A report of three cases. *International Journal of Gynecological Pathology*.

[4] Gupta RK, McHutchison AGR, Fauck R (1991). Liesegang rings in a needle aspirate from a breast cyst. *Acta Cytologica*.

[5] Gavin K, Banville N, Gibbons D, Quinn CM (2005). Liesegang rings in inflammatory breast lesions. *Journal of Clinical Pathology*.

[6] Raso DS, Greene WB, Finley JL (1998). Morphology and pathogenesis of Liesegang rings in cyst aspirates: report of two cases with ancillary studies. *Diagnostic Pathology*.

[7] Pegas KL, Edelweiss ML, Cambruzzi E (2010). Liesegang rings in xanthogranulomatous pyelonephritis: a case report. *Pathology Research International*.

[8] Sneige N, Dekmezian RH, Silva EG, Cartwright J, Ayala AG (1988). Pseudoparasitic Liesegang structures in perirenal hemorrhagic cysts. *American Journal of Clinical Pathology*.

